# Assessment of knowledge, attitudes, and practice regarding air pollution and health effects among general people: A multi-divisional cross-sectional study in Bangladesh

**DOI:** 10.1371/journal.pone.0305075

**Published:** 2024-06-11

**Authors:** Abu Bakkar Siddique, Md. Safaet Hossain Sujan, Sanjida Ahmed, Kifayat Sadmam Ishadi, Rafia Tasnim, Md. Saiful Islam, Md. Shakhaoat Hossain

**Affiliations:** Department of Public Health and Informatics, Jahangirnagar University, Savar, Dhaka, Bangladesh; University of Memphis, UNITED STATES

## Abstract

**Background:**

Bangladesh is one of the most densely populated countries in the world, with more than one-third of its people living in cities, and its air quality is among the worst in the world. The present study aimed to measure knowledge, attitudes and practice (KAP) towards air pollution and health effects among the general population living in the large cities in Bangladesh.

**Methods:**

A cross-sectional e-survey was conducted between May and July 2022 among eight divisions in Bangladesh. A convenience sampling technique was utilized to recruit a total of 1,603 participants (55.58% males; mean age: 23.84 ± 5.93 years). A semi-structured questionnaire including informed consent, socio-demographic information, as well as questions regarding knowledge (11-item), attitudes (7-item) and practice (11-item) towards air pollution, was used to conduct the survey. All analyses (descriptive statistics and regression analyses) were performed using STATA (Version 15.0) and SPSS (Version 26.0).

**Results:**

The mean scores of the knowledge, attitudes, and practice were 8.51 ± 2.01 (out of 11), 19.24 ± 1.56 (out of 21), and 12.65 ±5.93 (out of 22), respectively. The higher scores of knowledge, attitudes, and practice were significantly associated with several socio-demographic factors, including educational qualification, family type, residential division, cooking fuel type, etc.

**Conclusions:**

The present study found a fair level of knowledge and attitudes towards air pollution; however, the level of practice is not particularly noteworthy. The finding suggests the need to create more awareness among the general population to increase healthy practice to reduce the health effects of air pollution.

## Introduction

Air pollution poses a significant threat to human health and well-being. The South Asian region, including Bangladesh, experiences significant air pollution challenges due to factors like agricultural activities, increasing vehicle emissions, rapid urbanization, and population growth. It is crucial to acknowledge that these problems extend beyond national boundaries, intensifying their urgency [1,2]. Increased air pollution in developing countries (for example, China, Thailand, Malaysia, and Bangladesh) is mostly the result of fast economic and industrial expansion [3–5]. Air pollution is considered as 4^th^ leading risk factor for premature death globally [6]. Additionally, Air pollution was ranked as the second most significant global risk factor for non-communicable diseases and the second-highest risk factor for adverse health outcomes in East Asia [1]. According to the World Health Organization (WHO), ambient air pollution was responsible for 4.2 million deaths worldwide in 2021 [7]. Additionally, approximately 91% of global fatalities caused by air pollution are premature deaths, with the regions of WHO South Asia and Western Pacific exhibiting the highest rates [8].

Bangladesh is densely populated, with cities housing more than one-third of the population, and its air quality is among the worst in the world [9,10]. According to a global report on air pollution and health burden, Bangladesh documented a total of 173,500 deaths due to air pollution in 2019, which is over 50,000 more than the previous year, and it has been ranked as ninth among the top ten countries with the highest level of outdoor Ambient Particulate Matter (PM 2.5), which is very small at 2.5 micrometers in diameter or less, produced by all types of combustion common in urban and rural areas [11–13]. The most harmful particulate matter (PM 2.5), may cause significant health effects by entering deep into the respiratory tract and caused by 74,000 fatalities in Bangladesh [14]. In addition, 94,800 fatalities were caused by household air pollution from solid fuel, while the remaining deaths were brought on by exposure to ozone [15,16]. Therefore, air pollution is a major issue in our cities. Dhaka is the world’s most polluted city in terms of air pollution [17]. Between 2013 and 2015, PM 10 and PM 2.5 levels in Dhaka surpassed both yearly (50 gm/m^3^) and daily (150 gm/m^3^) national requirements [9]. Furthermore, thousands of Bangladeshis suffer from wheezing, asthma, coughing, chest pain, headaches, upper respiratory infections, and even death as a result of air pollution caused by vehicle exhaust, unplanned development and construction works, particulate matter, plastic wastes, brick kiln fumes, unsanitary hospital conditions, ashes, flames, and fires [18–20].

The government of Bangladesh is undertaking several programs and developing plans for future efforts to minimize air pollution [9]. Nevertheless, the effective execution of these strategies necessitates a comprehension of the knowledge, attitudes, and practice (KAP) regarding air pollution at both the individual and community levels. This is because KAP provides valuable insights and a genuine understanding of the population’s situation. A previous study conducted in Chandigarh, India, in 2022 reported that 79% of the people in that area lacked proper knowledge about air pollution, despite their concerns about it [21]. In such a scenario, people’s KAP towards air pollution is very important for government and policy makers to address all barriers for the implementations of air pollution protection plans and will help them to strengthen the pollution law and promote general people to maintain rules and regulations to protect themselves from ambient air pollution [22].

To date, there is no study among general people (the ordinary or common individuals who make up the population of Bangladesh) in Bangladesh investigating the KAP towards air pollution and its health effects. Consequently, the present study aimed to explore the KAP and associated socio-demographic factors towards air pollution, sex difference of each items, and health effects among general people across the major divisions in Bangladesh.

## Materials and methods

### Study design, participants, and procedure

The present study employed an e-survey-based cross-sectional study from May to July, 2022. The participants were enrolled using convenience and snowball sampling techniques. Each participant took approximately 8–10 minutes to complete the survey. Initially, 1,650 participants submitted the surveys. After removing incomplete responses, the final analysis included 1,603 responses. The data were gathered using a self-reported semi-structured questionnaire written in Bengali (the participant’s native language). A shareable link was generated after administering all of the questions. The survey link was shared on several community-based online forums to generate a large number of responses among people living in suburbs and urban areas.

A pilot test was carried out with 10 participants from the same population (target group) to determine the acceptability and transparency of the questionnaire. Following the pilot testing, a few minor adjustments were made to the questionnaire. These were not included in the final analysis. The first page of the questionnaire had an informed consent statement attached to it that explained the study’s objectives, procedures, and the participant’s right to decline the participation. Before starting the survey, participants were asked to obtain e-informed consent (i.e., *“Are you willing to participate in this study voluntarily and spontaneously*?*”*). If the respondent selected *"yes"*, they had access to the entire survey. If one selected *"no"*, a blank survey form was automatically submitted. The inclusion criteria of the participants included: ⅰ) being adults (≥ 18 years), ⅱ) being Bangladeshi residents, and ⅲ) having willingness to take part in the survey and consent. The participants below 18 years were extracted from the main analysis.

### Measures

#### Socio-demographic measures and determinants of air pollution

Socio-demographic information was gathered by asking questions about sex (male/ female), marital status (married/ unmarried/ divorced or widowed), education (below college [<11 grades]/ college [11–12 grades]/ university or above), monthly family income (<15,000 Bangladeshi Taka [BDT]/ 15,000–30,000 BDT / >30,000 BDT) [23] (1 USD ≈110.35 BDT in 28 October, 2023), occupation (student/ housewife/ employee/ businessman/ unemployed/ others), family type (nuclear/ joint), residence (rural/ urban), division (Dhaka/ Chattagram/ Rajshahi/ Khulna/ Barishal/ Sylhet/ Rangpur/ Mymensingh), and living periods (<2 years/ 2–5 years/ >5 years). In addition to the socio-demographic information, the fuel types used by the participants were also asked (wooden stick/ kerosene oil/ gas/ electricity).

#### Knowledge, attitudes, and practice measures

A total of 29 questions regarding knowledge (11-item), attitudes (7-item), and practice (11-item) towards air pollution and its health effects, were used in the present study which was adopted from previous literature through extensive literature review [9,24–30].

Eleven-item questions with three options (e.g., yes/ no/ don’t know) related to knowledge regarding air pollution and its health effects were asked to the participants (e.g., *“Does air pollution occur due to man-made causes*?*”*, *“Does air pollution cause more harm to the elderly and children*?*”*; see details in Table 2). During analysis, “yes” responses were coded as “1”; whereas “no” and “don’t know” responses were coded as “0”. The total score was obtained by summating the scores of all items and ranges from 0–11, with a higher score indicating a higher level of knowledge. In addition, sources of knowledge regarding air pollution and its health effects were also recorded from the participants.

To assess the attitudes towards air pollution and its health effects, seven questions were used with a three-point Likert scale (e.g., 1 = disagree, 2 = neutral, 3 = agree). Examples of such questions include: *“Does air pollution occur due to man-made causes*?*”*, *“Do you think air pollution can harm you*?*”* (see details in Table 4). The total score was obtained by summating the scores of all items and ranges from 7–21, with the higher score indicating a greater level of positive attitudes.

To document the practice status, the participants were asked eleven questions (e.g., *“Do you check air quality indicators*?*”*, *“Do you wear a mask when you go out at the time of air pollution*?; see details in Table 6). with three possible responses (e.g., never, sometimes, always). During analysis, “never” responses were coded as “0”, “sometimes” responses were coded as “1”, and “always” were coded as “2”. The total score was obtained by summating the scores of all items and ranges from 0–22, with a higher score indicating a higher level of practice.

### Statistical analysis

The data were analyzed using the Microsoft Excel (version 2019), Statistical Package for Social Sciences (SPSS version 25.0), and STATA (version 15.0). Cleaning, coding, and sorting were performed using the help of Microsoft Excel. Then, the excel file was imported in the SPSS software and the descriptive statistics (i.e., frequencies, percentages, means, standard deviations) were computed. Bivariate analyses (i.e., Chi-square test, Fisher’s Exact test) were also performed using the SPSS. Finally, bivariate and multivariable linear regression analyses were performed using the STATA including the total scores of knowledge, attitudes, and practice measures as dependent variables, and others (e.g., socio-demographics measures) as independent variables. A *p*-value less than 0.05 was regarded as significant for all sorts of analyses.

### Ethics

The study protocol was reviewed and approved by the Biosafety, Biosecurity and Ethical Clearance Committee, Jahangirnagar University, Savar, Dhaka-1342, Bangladesh [Ref No: BBEC, JU/ M-2022/4 (7)]. All procedures of the present study were conducted in accordance with human involving research guidelines (e.g., Helsinki declaration). Electronic written inform consent was obtained from each participant where the study’s procedures, objectives, and confidentiality about their information, etc., were clearly documented. The data were collected anonymously and analyzed using numerical codes.

## Results

### General characteristics of the participants

A total of 1,603 participants were included in the final analysis. Of them, majority were males (55.58%), unmarried (78.79%), and students (74.67%) (Table 1). Majority (74.49%) had university level of education, and 45.73% resided in Dhaka division. Most were from nuclear families (75.73%), and came from urban areas (81.78%). Majority reported that they resided in their respective cities for more than five years (69.49%). In addition, participants stated the following fuel types as their regular cooking: wooden sticks (18.53%), kerosene/ oil (1.19%), gas (75.05%), and electricity (5.24%).

**Table 1:** General characteristics of the participants (*N* = 1,603).

Variables	n (%)
**Age** (mean ± standard deviation)	23.84 ± 5.93 (years)
**Sex**	
Male	891 (55.58)
Female	712 (44.42)
**Marital status**	
Unmarried	1263 (78.79)
Married	331 (20.65)
Divorced/ widowed	9 (0.56)
**Education**	
Below college (< 11 grades)	89 (5.55)
College (11–12 grades)	320 (19.96)
University/ above	1194 (74.49)
**Monthly family income**	
< 15,000 BDT	368 (22.96)
15,000–30,000 BDT	556 (34.68)
> 30,000 BDT	679 (42.36)
**Occupation**	
Student	1197 (74.67)
Housewife	64 (3.99)
Employee	207 (12.91)
Businessman	63 (3.93)
Unemployed	59 (3.68)
Others	13 (0.81)
**Family type**	
Nuclear	1214 (75.73)
Joint	389 (24.27)
**Residence**	
Rural	292 (18.22)
Urban	1311 (81.78)
**Division**	
Dhaka	733 (45.73)
Chattogram	226 (14.1)
Rajshahi	115 (7.17)
Khulna	157 (9.79)
Barishal	133 (8.3)
Sylhet	33 (2.06)
Rangpur	145 (9.05)
Mymensingh	61 (3.81)
**Living periods**	
< 2 years	241 (15.03)
2–5 years	248 (15.47)
> 5 years	1114 (69.49)
**Fuel types**	
Wooden stick	297 (18.53)
Kerosene/ oil	19 (1.19)
Gas	1203 (75.05)
Electricity	84 (5.24)

### Knowledge regarding air pollution

The mean score of the knowledge items was 8.51 (SD = 2.01) out of 11, indicating an overall correct percentage of 77.36%. The distribution of each knowledge item and its sex difference are presented in Table 2. As per as multivariable linear regression analysis, the positively predicting factors of knowledge score included: ⅰ) having education bellow college level (< 11 grades) (β = 0.13, *p* < 0.001) in reference to ‘university/ above’, ⅱ) living with joint family (β = 0.07, *p* = 0.003) in reference to nuclear family, and ⅲ) residing in Barishal division (β = 0.06, *p* = 0.024) in reference to ‘Dhaka’ (Table 3). Whereas, the negatively predicting factors of knowledge score was fuel type (for wooden stick [β = -0.11, *p* = 0.031], and for gas [β = -0.21, *p* < 0.001] in reference to ‘electricity’) (Table 3).

**Table 2:** Distribution of each knowledge item and its sex difference.

**Variables**	**Overall** **n (%)**	**Male** **n (%)**	**Female** **n (%)**	***p*-value**
**Does air pollution occur due to man-made causes?**				
Yes	1560 (97.32)	869 (97.53)	691 (97.05)	0.295
No	26 (1.62)	11 (1.23)	15 (2.11)	
Don’t know	17 (1.06)	11 (1.23)	6 (0.84)	
**Does air pollution cause more harm to elderly people and babies?**				
Yes	1554 (96.94)	861 (96.63)	693 (97.33)	0.595
No	26 (1.62)	17 (1.91)	9 (1.26)	
Don’t know	23 (1.43)	13 (1.46)	10 (1.4)	
**Can fine dust particles (PM 10 and PM 2.5) penetrate deep into our lungs?**				
Yes	1380 (86.09)	766 (85.97)	614 (86.24)	0.012
No	28 (1.75)	23 (2.58)	5 (0.7)	
Don’t know	195 (12.16)	102 (11.45)	93 (13.06)	
**Can illness caused by exposure to fine particles (PM 10 and PM 2.5) cause miscarriage/ infant death?**				
Yes	1074 (67)	613 (68.8)	461 (64.75)	0.046
No	137 (8.55)	63 (7.07)	74 (10.39)	
Don’t know	392 (24.45)	215 (24.13)	177 (24.86)	
**Are people with heart and lung diseases at greater risk of developing disease/ illness caused by dust particles?**				
Yes	1519 (94.76)	839 (94.16)	680 (95.51)	0.483
No	35 (2.18)	22 (2.47)	13 (1.83)	
Don’t know	49 (3.06)	30 (3.37)	19 (2.67)	
**Does covering the face protect from air pollution?**				
Yes	866 (54.02)	480 (53.87)	386 (54.21)	0.981
No	630 (39.3)	352 (39.51)	278 (39.04)	
Don’t know	107 (6.67)	59 (6.62)	48 (6.74)	
**Do motor vehicles emit fine particulate matter (PM 2.5 and PM 10)?**				
Yes	1162 (72.49)	640 (71.83)	522 (73.31)	0.051
No	108 (6.74)	72 (8.08)	36 (5.06)	
Don’t know	333 (20.77)	179 (20.09)	154 (21.63)	
**Is the air in the city very polluted?**				
Yes	1544 (96.32)	853 (95.74)	691 (97.05)	0.360
No	41 (2.56)	27 (3.03)	14 (1.97)	
Don’t know	18 (1.12)	11 (1.23)	7 (0.98)	
**Are brick kilns most responsible for air pollution in the city?**				
Yes	916 (57.14)	487 (54.66)	429 (60.25)	0.001
No	537 (33.5)	332 (37.26)	205 (28.79)	
Don’t know	150 (9.36)	72 (8.08)	78 (10.96)	
**Since city air is very polluted, can staying indoors as much as possible protect us from air pollution?**				
Yes	677 (42.23)	386 (43.32)	291 (40.87)	0.577
No	839 (52.34)	456 (51.18)	383 (53.79)	
Don’t know	87 (5.43)	49 (5.5)	38 (5.34)	
**Does sitting in traffic jams cause more fine dust particles to enter our lungs?**				
Yes	1388 (86.59)	777 (87.21)	611 (85.81)	0.012
No	90 (5.61)	58 (6.51)	32 (4.49)	
Don’t know	125 (7.8)	56 (6.29)	69 (9.69)	

**Table 3:** Distribution of knowledge scores and regression analysis predicting knowledge.

Variables	Overall	*Bivariate regression analysis*					*Multivariable regression analysis*				
	Mean (SD)	B	SE	t	β	*p*-value	B	SE	t	β	*p*-value
**Age**		0.00	0.01	0.40	0.01	0.688	⸻	⸻	⸻	⸻	⸻
**Sex**											
Female	8.52 (1.96)	0.03	0.10	0.26	0.01	0.792	⸻	⸻	⸻	⸻	⸻
Male	8.5 (2.05)	Ref.									
**Marital status**											
Married	8.86 (2.03)	0.44	0.12	3.55	0.09	<0.001	0.23	0.16	1.44	0.05	0.149
Divorced/ widowed	8.22 (3.83)	-0.20	0.67	-0.29	-0.01	0.768	-0.81	0.68	-1.18	-0.02	0.236
Unmarried	8.42 (1.98)	Ref.					Ref.				
**Education**											
Below college (< 11 grades)	10.02 (2.11)	1.65	0.22	7.58	0.18	<0.001	1.17	0.25	4.64	0.13	**<0.001**
College (11–12 grades)	8.58 (2.05)	0.20	0.12	1.64	0.04	0.100	0.11	0.13	0.88	0.02	0.379
University/ above	8.38 (1.94)	Ref.					Ref.				
**Monthly family income**											
< 15,000 BDT	8.77 (2.1)	0.47	0.13	3.62	0.10	<0.001	0.20	0.14	1.45	0.04	0.146
15,000–30,000 BDT	8.59 (1.95)	0.29	0.11	2.54	0.07	0.011	0.19	0.12	1.67	0.05	0.095
> 30,000 BDT	8.3 (1.98)	Ref.					Ref.				
**Occupation**											
Housewife	9.03 (2.42)	0.62	0.26	2.40	0.06	0.016	0.12	0.29	0.41	0.01	0.682
Employee	8.62 (1.87)	0.21	0.15	1.39	0.03	0.166	0.09	0.18	0.49	0.01	0.623
Businessman	8.68 (2.33)	0.27	0.26	1.04	0.03	0.300	-0.23	0.28	-0.81	-0.02	0.417
Unemployed	9.05 (2.19)	0.64	0.27	2.38	0.06	0.017	0.28	0.27	1.04	0.02	0.300
Others	9.54 (3.26)	1.12	0.56	2.01	0.05	0.044	-0.18	0.59	-0.31	-0.01	0.759
Student	8.41 (1.95)	Ref.					Ref.				
**Family type**											
Joint	8.9 (2.14)	0.52	0.12	4.49	0.11	<0.001	0.35	0.12	2.96	0.07	**0.003**
Nuclear	8.38 (1.95)	Ref.					Ref.				
**Residence**											
Rural	8.87 (2.1)	0.45	0.13	3.44	0.09	0.001	-0.01	0.15	-0.04	<-0.01	0.966
Urban	8.43 (1.98)	Ref.					Ref.				
**Division**											
Chattogram	8.36 (1.95)	-0.01	0.15	-0.08	<-0.01	0.934	-0.08	0.15	-0.53	-0.01	0.599
Rajshahi	8.42 (2.01)	0.05	0.20	0.23	0.01	0.818	-0.11	0.20	-0.55	-0.01	0.585
Khulna	8.73 (1.98)	0.36	0.18	2.05	0.05	0.040	0.10	0.18	0.58	0.02	0.564
Barishal	8.92 (2.07)	0.55	0.19	2.93	0.08	0.003	0.42	0.19	2.26	0.06	**0.024**
Sylhet	8.45 (2.31)	0.08	0.36	0.23	0.01	0.815	-0.17	0.35	-0.47	-0.01	0.635
Rangpur	8.88 (2.08)	0.51	0.18	2.81	0.07	0.005	0.19	0.18	1.00	0.03	0.315
Mymensingh	8.56 (1.91)	0.19	0.27	0.70	0.02	0.485	0.08	0.26	0.31	0.01	0.757
Dhaka	8.37 (1.99)	Ref.					Ref.				
**Living periods**											
< 2 years	8.63 (1.96)	0.14	0.14	1.01	0.03	0.314	⸻	⸻	⸻	⸻	⸻
2–5 years	8.47 (2.07)	-0.02	0.14	-0.16	<-0.01	0.869	⸻	⸻	⸻	⸻	⸻
> 5 years	8.49 (2.01)	Ref.					Ref.				
**Fuel types**											
Wooden stick	9.14 (2.13)	-0.18	0.24	-0.74	-0.03	0.460	-0.57	0.26	-2.16	-0.11	**0.031**
Kerosene/ oil	9.21 (2.82)	-0.11	0.50	-0.22	-0.01	0.825	-0.57	0.51	-1.10	-0.03	0.269
Gas	8.29 (1.92)	-1.04	0.22	-4.65	-0.22	<0.001	-0.98	0.22	-4.35	-0.21	**<0.001**
Electricity	9.32 (1.9)	Ref.					Ref.				

**Fig 1:**
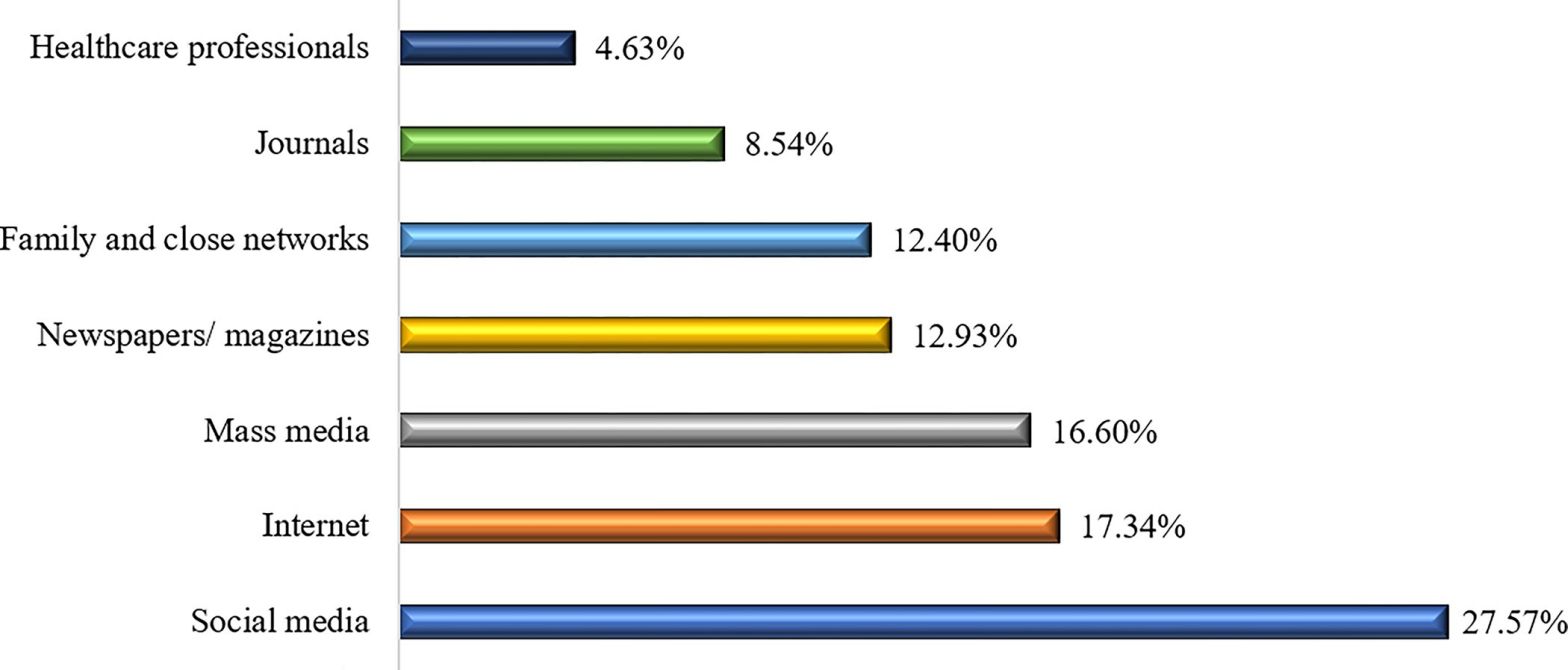
Source of knowledge regarding air pollution.

Fig 1 demonstrates the sources of knowledge regarding air pollution. A substantial proportion reported the main sources of their knowledge as social media (27.57%), internet (17.34%), and mass media (16.60%).

### Attitudes regarding air pollution

The mean score of the attitudes items was 19.24 (SD = 1.56) out of 21, indicating an overall correct percentage of 91.61%. The distribution of each attitudes item and its sex difference are presented in Table 4. As per as multivariable linear regression analysis, the positively predicting factors of attitudes score included: ⅰ) having education bellow college level (< 11 grades) (β = 0.10, *p* < 0.001) in reference to ‘university/ above’, ⅱ) having monthly income < 15,000 BDT (β = 0.07, *p* = 0.016) in reference to ‘> 30,000 BDT’, ⅲ) living with joint family (β = 0.05, *p* = 0.040), and ⅳ) residence (for Chattogram [β = 0.06, *p* = 0.029], and for Rangpur division [β = 0.06, *p* = 0.027] in reference to ‘Dhaka’) (Table 5). Whereas, the negatively predicting factors of attitudes score was having fewer living periods in the current residence (for 2–5 years [β = -0.06, *p* = 0.016] in reference to ‘> 5 years’) (Table 5).

**Table 4:** Distribution of each attitudes item and its sex difference.

Variables	Overall n (%)	Male n (%)	Female n (%)	*p*-value
**Do you think air pollution is a serious problem?**				
Disagree	5 (0.31)	2 (0.22)	3 (0.42)	0.266*
Undecided	34 (2.12)	23 (2.58)	11 (1.54)	
Agree	1564 (97.57)	866 (97.19)	698 (98.03)	
**Do you think air pollution can harm you?**				
Disagree	9 (0.56)	6 (0.67)	3 (0.42)	0.190*
Undecided	57 (3.56)	38 (4.26)	19 (2.67)	
Agree	1537 (95.88)	847 (95.06)	690 (96.91)	
**Do you think anyone in your family is likely to be harmed by air pollution?**				
Disagree	25 (1.56)	15 (1.68)	10 (1.4)	0.462
Undecided	102 (6.36)	51 (5.72)	51 (7.16)	
Agree	1476 (92.08)	825 (92.59)	651 (91.43)	
**Do you think the air quality in your city is good enough?**				
Disagree	571 (35.62)	297 (33.33)	274 (38.48)	0.101
Undecided	553 (34.5)	318 (35.69)	235 (33.01)	
Agree	479 (29.88)	276 (30.98)	203 (28.51)	
**Do you think our awareness/public awareness will help to reduce air pollution?**				
Disagree	31 (1.93)	19 (2.13)	12 (1.69)	0.001
Undecided	117 (7.3)	84 (9.43)	33 (4.63)	
Agree	1455 (90.77)	788 (88.44)	667 (93.68)	
**Do you think it is the responsibility of all citizens to keep the environment clean in your city?**				
Disagree	8 (0.5)	4 (0.45)	4 (0.56)	0.442*
Undecided	52 (3.24)	33 (3.7)	19 (2.67)	
Agree	1543 (96.26)	854 (95.85)	689 (96.77)	
**If you were asked to lessen the use of your own car to reduce air pollution, would you agree?**				
Disagree	129 (8.05)	78 (8.75)	51 (7.16)	0.498
Undecided	347 (21.65)	193 (21.66)	154 (21.63)	
Agree	1127 (70.31)	620 (69.58)	507 (71.21)	

Note: *Fisher’s Exact Test.

**Table 5:** Distribution of attitudes scores, and regression analysis predicting attitudes.

Variables	Overall	*Bivariate regression analysis*					*Multivariable regression analysis*				
	Mean (SD)	B	SE	t	β	*p*-value	B	SE	t	β	*p*-value
**Age**		0.00	0.01	-0.47	-0.01	0.638	⸻	⸻	⸻	⸻	⸻
**Sex**											
Female	19.26 (1.5)	0.04	0.08	0.50	0.01	0.615	⸻	⸻	⸻	⸻	⸻
Male	19.22 (1.61)	Ref.									
**Marital status**											
Married	19.43 (1.51)	0.23	0.10	2.39	0.06	0.017	0.14	0.12	1.11	0.04	0.267
Divorced/ widowed	19 (2.5)	-0.20	0.52	-0.37	-0.01	0.708	-0.78	0.53	-1.46	-0.04	0.144
Unmarried	19.2 (1.57)	Ref.					Ref.				
**Education**											
Below college (< 11 grades)	20.21 (1.57)	1.08	0.17	6.39	0.16	<0.001	0.71	0.20	3.57	0.10	**<0.001**
College (11–12 grades)	19.39 (1.49)	0.26	0.10	2.69	0.07	0.007	0.17	0.10	1.73	0.04	0.084
University/ above	19.13 (1.55)	Ref.					Ref.				
**Monthly family income**											
< 15,000 BDT	19.51 (1.61)	0.46	0.10	4.57	0.12	<0.001	0.26	0.11	2.40	0.07	**0.016**
15,000–30,000 BDT	19.29 (1.39)	0.24	0.09	2.67	0.07	0.008	0.16	0.09	1.72	0.04	0.085
> 30,000 BDT	19.05 (1.64)	Ref.					Ref.				
**Occupation**											
Housewife	19.73 (1.29)	0.54	0.20	2.71	0.07	0.007	0.25	0.22	1.13	0.03	0.258
Employee	19.22 (1.55)	0.03	0.12	0.25	0.01	0.803	0.00	0.14	0.03	<0.01	0.976
Businessman	19.06 (2.33)	-0.13	0.20	-0.64	-0.02	0.520	-0.43	0.22	-1.96	-0.05	0.050
Unemployed	19.73 (1.52)	0.54	0.21	2.58	0.06	0.010	0.32	0.21	1.52	0.03	0.130
Others	20.31 (1.97)	1.12	0.43	2.57	0.06	0.010	0.30	0.46	0.64	0.02	0.522
Student	19.19 (1.52)	Ref.					Ref.				
**Family type**											
Joint	19.49 (1.68)	0.33	0.09	3.59	0.09	<0.001	0.19	0.09	2.06	0.05	**0.040**
Nuclear	19.16 (1.52)	Ref.					Ref.				
**Residence**											
Rural	19.53 (1.65)	0.35	0.10	3.50	0.09	<0.001	-0.01	0.12	-0.12	<-0.01	0.905
Urban	19.18 (1.54)	Ref.					Ref.				
**Division**											
Chattogram	19.37 (1.31)	0.26	0.12	2.21	0.06	0.028	0.26	0.12	2.18	0.06	**0.029**
Rajshahi	19.23 (1.69)	0.12	0.16	0.80	0.02	0.426	0.06	0.16	0.39	0.01	0.694
Khulna	19.11 (1.58)	0.00	0.14	0.03	<0.01	0.976	-0.10	0.14	-0.73	-0.01	0.468
Barishal	19.37 (1.86)	0.26	0.15	1.76	0.05	0.079	0.19	0.15	1.32	0.03	0.188
Sylhet	19.67 (1.47)	0.56	0.28	2.01	0.05	0.045	0.43	0.28	1.58	0.04	0.114
Rangpur	19.61 (1.8)	0.50	0.14	3.56	0.09	<0.001	0.32	0.15	2.21	0.06	**0.027**
Mymensingh	19.3 (1.24)	0.19	0.21	0.89	0.02	0.374	0.16	0.21	0.79	0.02	0.432
Dhaka	19.11 (1.51)	Ref.					Ref.				
**Living periods**											
< 2 years	19.3 (1.44)	0.03	0.11	0.24	0.01	0.809	-0.10	0.11	-0.91	-0.02	0.363
2–5 years	19.05 (1.67)	-0.22	0.11	-2.00	-0.05	0.045	-0.27	0.11	-2.41	-0.06	**0.016**
> 5 years	19.27 (1.56)	Ref.					Ref.				
**Fuel types**											
Wooden stick	19.71 (1.7)	0.45	0.19	2.35	0.11	0.019	0.06	0.21	0.27	0.01	0.791
Kerosene/ oil	19.47 (2.12)	0.21	0.39	0.54	0.01	0.590	-0.22	0.40	-0.55	-0.02	0.581
Gas	19.12 (1.48)	-0.14	0.18	-0.80	-0.04	0.421	-0.19	0.18	-1.08	-0.05	0.281
Electricity	19.26 (1.81)	Ref.					Ref.				

### Practice regarding air pollution

The mean score of the practice items was 12.65 (SD = 5.93) out of 22, indicating an overall correct percentage of 57.5%. The distribution of each practice item and its sex difference are presented in Table 6. As per as multivariable linear regression analysis, the positively predicting factors of practice score included: ⅰ) having monthly income < 15,000 BDT (β = 0.06, *p* = 0.030) in reference to ‘> 30,000 BDT’, ⅱ) living with joint family (β = 0.08, *p* = 0.001), and ⅲ) residence (for Barishal [β = 0.10, *p* < 0.001], for Sylhet [β = 0.07, *p* = 0.003], and for Rangpur division [β = 0.06, *p* = 0.016] in reference to ‘Dhaka’) (Table 7). Whereas, the negatively predicting factors of practice score included: ⅰ) education (for college [11–12 grades] [β = -0.23, *p* < 0.001], and for university/ above [β = -0.34, *p* < 0.001] in reference to ‘Below college [< 11 grades]’), and ⅱ) fuel type (for gas [β = -0.16, *p* = 0.001] in reference to ‘electricity’ (Table 7).

**Table 6:** Distribution of each practice item and its sex difference.

Variables	Overall n (%)	Male n (%)	Female n (%)	*p*-value
**Do you check air quality indicators?**				
Never	533 (33.25)	255 (28.62)	278 (39.04)	<0.001
Sometime	699 (43.61)	419 (47.03)	280 (39.33)	
Always	371 (23.14)	217 (24.35)	154 (21.63)	
**Do you wear a mask when you go out at the time of air pollution?**				
Never	93 (5.8)	66 (7.41)	27 (3.79)	<0.001
Sometime	690 (43.04)	437 (49.05)	253 (35.53)	
Always	820 (51.15)	388 (43.55)	432 (60.67)	
**Have you reduced the amount/ number of window openings to protect against air pollution?**				
Never	349 (21.77)	196 (22)	153 (21.49)	0.226
Sometime	693 (43.23)	370 (41.53)	323 (45.37)	
Always	561 (35)	325 (36.48)	236 (33.15)	
**Have you reduced your outdoor activities to avoid dust/ air pollution?**				
Never	720 (44.92)	376 (42.2)	344 (48.31)	0.045
Sometime	469 (29.26)	270 (30.3)	199 (27.95)	
Always	414 (25.83)	245 (27.5)	169 (23.74)	
**Do you stop using roads/ highways to avoid dust/ air pollution?**				
Never	684 (42.67)	355 (39.84)	329 (46.21)	0.012
Sometime	496 (30.94)	278 (31.2)	218 (30.62)	
Always	423 (26.39)	258 (28.96)	165 (23.17)	
**Does your family tell you about particulate matter and its harmful effects?**				
Never	251 (15.66)	151 (16.95)	100 (14.04)	0.074
Sometime	625 (38.99)	357 (40.07)	268 (37.64)	
Always	727 (45.35)	383 (42.99)	344 (48.31)	
**Do your neighbors and friends tell you about particulate matter and its harmful effects?**				
Never	338 (21.09)	176 (19.75)	162 (22.75)	0.291
Sometime	658 (41.05)	377 (42.31)	281 (39.47)	
Always	607 (37.87)	338 (37.93)	269 (37.78)	
**Does your family ask you to take protective measures against air pollution?**				
Never	205 (12.79)	119 (13.36)	86 (12.08)	0.460
Sometime	565 (35.25)	321 (36.03)	244 (34.27)	
Always	833 (51.97)	451 (50.62)	382 (53.65)	
**Do your friends and family ask you to take protective measures against air pollution?**				
Never	297 (18.53)	151 (16.95)	146 (20.51)	0.165
Sometime	642 (40.05)	359 (40.29)	283 (39.75)	
Always	664 (41.42)	381 (42.76)	283 (39.75)	
**Have you taken/ learned information from any environmental scientist/ expert about the harmful effects of fine dust particles?**				
Never	167 (10.42)	80 (8.98)	87 (12.22)	0.102
Sometime	636 (39.68)	356 (39.96)	280 (39.33)	
Always	800 (49.91)	455 (51.07)	345 (48.46)	
**Have you learned about air pollution and its remedies from health volunteers?**				
Never	553 (34.5)	284 (31.87)	269 (37.78)	0.008
Sometime	434 (27.07)	236 (26.49)	198 (27.81)	
Always	616 (38.43)	371 (41.64)	245 (34.41)	

**Table 7:** Distribution of practice scores, and regression analysis predicting practice.

Variables	Overall	*Bivariate regression analysis*					*Multivariable regression analysis*				
	Mean (SD)	B	SE	t	β	*p*-value	B	SE	t	β	*p*-value
**Age**		-0.01	0.03	-0.34	-0.01	0.735	⸻	⸻	⸻	⸻	⸻
**Sex**											
Female	12.46 (5.93)	-0.33	0.30	-1.12	-0.03	0.263	⸻	⸻	⸻	⸻	⸻
Male	12.8 (5.94)	Ref.									
**Marital status**											
Married	14.01 (6.23)	1.72	0.36	4.73	0.12	<0.001	0.77	0.45	1.70	0.05	0.088
Divorced/ widowed	13.56 (8.72)	1.27	1.97	0.64	0.02	0.521	-2.00	1.95	-1.03	-0.03	0.305
Unmarried	12.29 (5.78)	Ref.					Ref.				
**Education**											
College (11–12 grades)	13.56 (6.16)	6.84	0.63	10.89	0.26	<0.001	-3.44	0.75	-4.61	-0.23	**<0.001**
University/ above	11.95 (5.57)	1.61	0.36	4.46	0.11	<0.001	-4.63	0.73	-6.39	-0.34	**<0.001**
Below college (< 11 grades)	18.79 (5.97)	Ref.					Ref.				
**Monthly family income**											
< 15,000 BDT	13.87 (6.32)	1.98	0.38	5.20	0.14	<0.001	0.87	0.40	2.17	0.06	**0.030**
15,000–30,000 BDT	12.77 (5.86)	0.87	0.34	2.59	0.07	0.010	0.45	0.33	1.36	0.04	0.173
> 30,000 BDT	11.89 (5.66)	Ref.					Ref.				
**Occupation**											
Housewife	15.45 (6.14)	3.20	0.75	4.25	0.11	<0.001	1.09	0.82	1.34	0.40	0.182
Employee	12.73 (5.5)	0.48	0.44	1.08	0.03	0.280	0.23	0.50	0.45	0.01	0.654
Businessman	14.02 (6.79)	1.76	0.76	2.32	0.06	0.020	-0.23	0.80	-0.29	-0.01	0.774
Unemployed	14.53 (6.99)	2.27	0.78	2.90	0.07	0.004	1.00	0.77	1.30	0.03	0.194
Others	18.62 (6.95)	6.36	1.64	3.89	0.10	<0.001	1.23	1.69	0.73	0.02	0.467
Student	12.26 (5.79)	Ref.					Ref.				
**Family type**											
Joint	14.12 (6.27)	1.95	0.34	5.68	0.14	<0.001	1.15	0.34	3.38	0.08	**0.001**
Nuclear	12.18 (5.75)	Ref.					Ref.				
**Residence**											
Rural	13.93 (6.27)	1.56	0.38	4.09	0.10	<0.001	0.44	0.43	1.02	0.03	0.309
Urban	12.37 (5.82)	Ref.					Ref.				
**Division**											
Chattogram	12.55 (5.7)	0.70	0.45	1.57	0.04	0.116	0.49	0.43	1.14	0.03	0.254
Rajshahi	12.91 (5.96)	1.07	0.59	1.81	0.05	0.070	0.45	0.57	0.79	0.02	0.431
Khulna	13.02 (5.57)	1.17	0.52	2.28	0.06	0.023	0.34	0.51	0.67	0.02	0.502
Barishal	14.51 (6.13)	2.66	0.55	4.83	0.12	<0.001	2.13	0.54	3.99	0.10	**<0.001**
Sylhet	15.7 (6.3)	3.85	1.04	3.69	0.09	<0.001	2.95	1.01	2.94	0.07	**0.003**
Rangpur	14.3 (6.23)	2.46	0.53	4.62	0.12	<0.001	1.28	0.53	2.42	0.06	**0.016**
Mymensingh	11.61 (5.29)	-0.24	0.78	-0.31	-0.01	0.758	-0.55	0.75	-0.74	-0.02	0.462
Dhaka	11.85 (5.84)	Ref.					Ref.				
**Living periods**											
< 2 years	12.86 (6.17)	0.19	0.42	0.45	0.01	0.652	⸻	⸻	⸻	⸻	⸻
2–5 years	12.37 (5.45)	-0.30	0.42	-0.72	-0.02	0.469	⸻	⸻	⸻	⸻	⸻
> 5 years	12.67 (5.99)	Ref.					Ref.				
**Fuel types**											
Wooden stick	15.12 (6.47)	0.94	0.72	1.31	0.06	0.189	-0.76	0.76	-1.00	-0.05	0.318
Kerosene/ oil	15.89 (6.4)	1.72	1.47	1.17	0.03	0.243	-1.01	1.48	-0.68	-0.02	0.495
Gas	11.88 (5.59)	-2.30	0.65	-3.52	-0.17	<0.001	-2.18	0.64	-3.39	-0.16	**0.001**
Electricity	14.18 (5.78)	Ref.					Ref.				

## Discussion

Air pollution has recently raised as a burning environmental concern in Asia and other regions of the globe [31]. People living in the major cities of Bangladesh are at risk due to its higher levels of air pollution compared to the WHO-recommended thresholds [9]. Thus, the present study assessed the knowledge, attitudes and practice among the general population residing in the cities all over Bangladesh. The findings demonstrated favorable knowledge and attitudes but less practice levels regarding air pollution and its health effects; and associated factors of knowledge, attitudes and practice.

In the present study, the knowledge about air pollution varies significantly among education level, type of family, division of residence and type of fuel usage. The respondents who attended bellow college-level education (<11 grade) perceived higher knowledge regarding air pollution than college and university-level participants. This finding aligned with prior study, where the studied population with lower educational levels had more knowledge and perception regarding air pollution than those with higher educational levels [32,33]. At the secondary level of education in Bangladesh, various subject areas cover environmental concepts [34] which might be helpful for gaining knowledge about air pollution among people who received at least secondary level education. In contrast with the finding, a previous study showed a linear relationship between increased education and increased knowledge awareness regarding air pollution [30]. Further investigation is needed to clarify this controversial association between knowledge on air pollution and educational level.

The study showed that participants who belonged to joint family had higher knowledge score on air pollution than those from nuclear family. Also, their attitudes and practice found to be consistent with their knowledge level in the study. Supporting to the finding, previous studies suggested that, family plays an influential role in shaping ideation of the effects of air pollution through sharing information about air pollution, which creates positive impact on the knowledge growth among family members [9,35].

Among eight divisions, the respondents residing in Barishal had better knowledge concerning air pollution in the present study. The lower levels of air pollution in Barishal foster a well-informed population due to reduced exposure, proactive educational initiatives, government policies, community engagement, access to resources, and media influence. The attitudes towards air pollution were found to be higher among the participants from Rangpur and Chattogram divisions as well as the protective practices were found higher among the respondents from Sylhet, Rangpur and Barishal divisions. The variation among different divisions regarding knowledge, attitudes and practice towards air pollution could be due to the imagination, place identity, exposure experience, socio-demographical condition, seasonal variation, etc. A study from Beijing found that place identity and people’s imagination during the survey can affect participant’s perception about air quality [25]. Another study stated that, education, income level as well as previous suffering experiences from air pollution differs from place to place which affect people’s knowledge and understanding regarding air pollution [30,35].

In the present study, knowledge of air pollution and health effects was significantly lower among the study participants who used natural gas and wooden stick as cooking fuel compared to those who used electricity which echoed the findings from an earlier study [27]. This finding is consistent with a prior study which observed that people who use electricity as cooking energy had better knowledge about the detrimental effects of biomass fuel [27] and another studies showed that socioeconomic circumstances, ethno-environmental tradition, and local community all play significant roles in how well people are knowledgeable about choosing various cooking fuel types [36,37]. The possible explanation for low level knowledge might be due to lack of information regarding environmental effects of different fuel types where the local community can play an important role. Low level knowledge and lack of protective practices about air pollution among the gas users aligned together in this study. A prior study found that decisions from family members had an influence on changing the fuel types [38] which might be attributable to the poor protective practice during air pollution among gas users.

Social media, internet, mass media, newspaper/ magazine, family close network, and journals were the common source of obtaining information about air pollution. Social media was reported as the most frequently used source of gaining knowledge regarding air pollution which is consistent with an earlier study from Muscat [26]. Prior study depicted that people prefer having information with proper audio-visual capacity and suggested to utilize digital platforms which might be helpful for gaining better insights into air pollution [26,30].

Respondents with below college level education showed positive attitudes, which is coherent with their knowledge level regarding air pollution. Interestingly, this study found an inverse association between increased education level and protective practices among the participants which contradicts findings from Ningbo, China [30]. Another study showed no significant association between educational level and level of concern about air pollution [39]. Possibly, this observation could be due to internal factors (e.g., knowledge, attitude, intention) and external factors (e.g., social support and environment) which influence people to practice certain behaviors [40]. Further research should be done for the clarification of these inconsistent findings.

In line with the previous study conducted in the United States [41], our study revealed that participants with lower income (<15,000 BDT) had more receptive attitudes and careful practices regarding air quality than high income population. Results from an earlier study showed that the number of working people are higher among low-income population [36], and possibly for this, they need to go outside more and experience air pollution than high-income population which might be an explanation for their responsive attitude and protective practices regarding air pollution.

Participants who had been living in a certain place for two-three years had lessened attitudes towards air pollution than those who had been living for more than five years. Similar to our study, a previous study found that level of attitude towards air pollution seemed to be associated with the living period within a certain place [24]. Air quality in a certain place served as a standard point for making perception regarding air pollution where people lived for a longer period of time [25]. With reference to this, our study population had a lower level of attitudes towards air pollution since they didn’t live longer within certain community to perceive a better level of attitudes towards air pollution.

### Limitations

There are some limitations in the present study. Firstly, this was a cross-sectional study and the causality of factors could not be established. A longitudinal study would be helpful in this regard. Secondly, the study employed an online-based self-reporting method, which could have been influenced by a variety of biases (e.g., social desirability, and memory recall). Thirdly, the findings may not be considered representative due to the online data, convenience sampling technique, and largest proportion of younger age group. A future study with more representative samples using face-to-face interviews and random sampling is recommended to overcome the limitations.

## Conclusions

The present study investigated the knowledge, attitudes and practice level regarding air pollution and its health effects among the general people of eight divisions in Bangladesh, as well as explored their associated factors. The current study contributes to the field of environmental health research focusing on air pollution which showed that the level of practice to reduce air pollution exposure is not sufficient. The findings of this study expect to draw attention from policy makers, city mayors and responsible authorities to take necessary interventions and awareness raising activities on air pollution throughout the multicity of Bangladesh. Findings from this study also suggest that utilizing popular media with proper caution (social media, mass media, etc.) can be helpful for policy makers in designing and implementing effective channels for promoting education on air pollution exposure and health effects. Meanwhile, further research is needed for assessing knowledge, attitudes and practice about air pollution among different study groups for underpinning the current study findings.

## Supporting information

S1 Dataset (XLSX)
